# Plant Biology Research: What Is Next?

**DOI:** 10.3389/fpls.2021.749104

**Published:** 2021-09-30

**Authors:** Anna N. Stepanova

**Affiliations:** Department of Plant and Microbial Biology, Program in Genetics, North Carolina State University, Raleigh, NC, United States

**Keywords:** plant biology, plant physiology, synthetic biology, translational research, data reproducibility

Plant biology is a key area of science that bears major weight in the mankind's ongoing and future efforts to combat the consequences of global warming, climate change, pollution, and population growth. An in-depth understanding of plant physiology is paramount to our ability to optimize current agricultural practices, to develop new crop varieties, or to implement biotechnological innovations in agriculture. The next-generation cultivars would have to withstand environmental contamination and a wider range of growth temperatures, soil nutrients and moisture levels and effectively deal with growing pathogen pressures to continue to yield well in even suboptimal conditions.

What are the next big questions in plant physiology, and plant biology in general, and what avenues of research should we be investigating and training students in for the next decade? As a plant scientist surrounded by like-minded individuals, I hear a lot of ideas that over time turn into buzz words, such as plant resilience, genotype-to-phenotype, data science, systems biology, biosensing, synthetic biology, neural networks, robustness, interdisciplinary training, new tool development, modeling, etc. What does it all mean and what are the main challenges that we should all be working on solving? Herein, I present my personal perspective on what the immediate questions and the biggest longer-term issues in plant science are. I suggest some themes and directions for future research in plant biology, some relatively obvious and some potentially unique, having been shaped by my own professional interests, experiences and the background in plant molecular genetics and physiology.

## Integration, Packaging, Visualization and Interpretation of Existing OMICS and Genetic Data

For the past three decades, a lot of emphasis has been made on a small set of plant model organisms, primarily on Arabidopsis. There is no other plant on earth we know as much about as we do about this mustard weed. One clear need in the area of plant sciences is to make sense of the vast amount of descriptive phenotypic data that have been generated for this species and a handful of others—the transcriptome, metabolome, proteome, phenome, interactome, etc.—and the amazing genetic resources that have been built: mutants, transgenic lines and natural accession germplasm collections, tools and protocols, genomic sequences and other resources (Koorneef and Meinke, 2010). Now, how do we organize these data into a series of integrated, comprehensive, user-friendly, cross-communicating databases that are easily accessible, searchable, trackable, and visual, with data that are downloadable and compatible with comparative analyses? How do we display the available data at a variety of scales, from the subcellular to the organismal and population level—think Google Earth but for an ecosystem or an agricultural field that allows you to zoom in and out to see the overview and the closeup—perhaps, by integrating and expanding existing initiative likes Plant Cell Atlas and ePlant (Waese et al., 2017; Rhee et al., 2019)? With the genome sequences of these select organisms in hand, often of multiple accessions of each, what can we learn about the genotype-to-phenotype relations? How can we use that knowledge to extrapolate the rules or patterns we discover in model organisms to species for which we have no experimental data beyond possibly a draft-quality genomic sequence and a few fragmentary phenotypic datasets? In other words, can the data obtained in reference organisms be leveraged to infer useful information relevant to a wide range of species of agricultural, ecological or, perhaps, ethnobotanical importance? Let's look into some examples of that.

## Translational Research: Moving Foundational Discoveries From Models to Crops

It comes as no surprise that for the past 10–20 years the emphasis has been gradually shifting from Arabidopsis to non-model organisms, including crops and rare plant species. The key reason for that is the pressing need to move fast on crop improvement and plant conservation in light of the worlds' fast-growing population, climate change, pollution, habitat and agricultural land loss, and ever-increasing pathogen pressures. This shift of research focus is also steered by changing governmental policies and funders' priorities. To make the transition to studying crops and other non-models as smooth as possible, robust computational pipelines are needed that produce high-quality genome assemblies from combinations of short- and long-read sequences. In this regard, tackling the much more complex genomes of polyploid species presents an even greater challenge. With the genome sequences and high-quality assembles on hand, orthologous genes that have previously been studied only in reference organisms need to be tested for function in candidate processes in the non-model species of interest to determine what aspects of their function are conserved and what features are divergent. The key bottleneck in this process is, of course, the recalcitrance of many non-models to genetic transformation and plant regeneration (Anjanappa and Gruissem, 2021). Thus, a major effort would need to be invested into new method development to improve the plant *in vitro* culturing, genetic transformation and regeneration pipelines, with the ectopic activation of morphogenesis genes like *BABY BOOM, WUSCHEL, LEAFY COTYLEDON1* and *2*, and several others holding major promise for boosting the regeneration efficiency of otherwise recalcitrant plant species and cultivars (Gordon-Kamm et al., 2019). Further optimization of genome editing technologies, including classical gene disruption through indels as well as more targeted gene edits *via* base- and prime-editing or homologous-recombination-based methods, should enable highly tailored manipulation of genes of interest. The foundational knowledge gained in both model and non-model organisms can then be leveraged by applied plant biologists and environmentalists in crop improvement and plant conservation.

## Interpreting the Code

One aspect of experimental research we have become good at over the past 10 years is genome and transcriptome sequencing. The current challenge is to learn to infer what the sequence tells us about what a gene does and how it is regulated based on the code alone. Can we look at gene's genomic sequence and infer not only the gene function, but also the different levels of gene regulation, all from just the sequence without any additional experimentation? To elaborate on that distinction between function and regulation, we can already infer the likely function of an orthologous gene in a crop (previously studied in another species) based on the degree of conservation of its genomic sequence, and deduce, for instance, an enzymatic reaction a protein may catalyze, or a DNA element a transcription factor may bind, or a specific ion the channel may transport, or an array of ligands or other molecules a protein may interact with. What we cannot yet reliably do is to predict based on the gene sequence alone when and where the gene is transcribed and what environmental or developmental stimuli alter its expression, how stable its transcript is, what splicing patterns the transcript has in specific cell types or conditions, or what factors dictate these patterns, or how well the transcript is translated, how the protein folds, where in the cell the protein is targeted, what its half-life is, and so on. Can we someday look at the gene sequence and predict whether the gene is essential or what organ or tissues will be affected in the loss- or gain-of-function mutant, and what phenotype the mutant will show, all without having to run an experiment? Once we learn to do that for a diploid model plant, can the knowledge be translated to polyploids that may have a greater level of gene redundancy and potentially more cases of neofunctionalization? How do we gain that extraordinary power?

One of the critical components of the inferring-the-function or genotype-to-phenotype challenge will involve machine learning and neural network models, with the size and quality of the training datasets presenting as the likely bottleneck that would determine the accuracy of neural networks' predictions (Ching et al., 2018). While the role of computational biologists in this endeavor would be to develop new algorithms or adapt existing pipelines and test the models, the irreplaceable function of experimental plant biologists in this effort will be to generate the most complete and robust datasets for model training. This inevitably brings us to the next big theme, data quality.

## Data Quality: Standardization, Reliability, Robustness and Tracking

As experimental scientists, most if not all of us have had the negative experience of not being able to reproduce an important result (sometimes even our own) or confirm the identity of a material someone has shared with us (e.g., a strain, a plasmid, or a seed stock from a colleague or another lab). Issues with biological variation (e.g., differences in germination between seed batches), small sample size (due to prohibitive cost, time or material constraints, or other limitations), human error (suboptimal labeling nomenclature, poor tracking, inadequate record keeping, substandard experimental design, miscalculation, personnel changes, or outright sloppiness) or malfunctioning instrumentation (in many cases, due to the lack of funding or time to upkeep or upgrade the equipment) can all contribute to the limited reproducibility of experimental data or sample mix-up. Rarely is the wrongdoing intentional, but the consequences of these errors can be enormous. What can we do to minimize mistakes, standardize internal lab protocols and record keeping, and ultimately improve the reproducibility of published data? I would support a universal funder's mandate for detailed electronic note keeping (much like private companies require), automatic data backups and regular equipment upgrades, meticulous planning before an experiment is run (including developing a comprehensive sample labeling nomenclature, beyond the common 1, 2, 3), inclusion of universal controls (e.g., Arabidopsis Columbia accession included in every Arabidopsis experiment irrespective of what other germplasm is being tested), extensive sample replication, validation of the results at multiple steps in the process (like Sanger sequencing of construct intermediates), and other common-sense but often time-consuming practices (such as regrowing all genotypes side by side and using fresh seed stocks in an experiment to minimize seed batch effects, or resequencing every construct before donating it to the stock center or sharing it with others).

A different yet related constraint we often encounter in plant sciences is the inability to track and/or obtain the materials or datasets reported by other research groups or oftentimes even by prior members of one's own lab. To ensure the long-term availability and unrestricted access to published constructs, germplasm, omics datasets and other resources generated by the public sector, funding agencies should make it mandatory for all materials and data to be deposited in relevant stock centers, sequence repositories, etc. immediately upon publication. I often wonder whether this practice could be encouraged if one's scientific productivity and impact were to be evaluated not only by the number of papers published, but also by the number of stocks or datasets deposited and their usage by the community (e.g., the frequency of stock orders or data downloads). Publishers, on the other hand, should fully enforce the old rules that all submitted manuscripts must adhere to the established guidelines for proper scientific nomenclature (e.g., gene accession numbers, mutant names, or chemical structures) and include community access codes (e.g., gene identifiers, mutant stock numbers, Genbank accession codes, etc.) and detailed annotations for all materials and data utilized or generated in a study, with the compliance being a prerequisite for publication. These simple steps would reduce ambiguities, facilitate resource tracking, and make published materials and datasets universally available.

The extra effort invested into careful experiment planning, execution, record keeping, and making published materials and datasets trackable and accessible will undoubtedly lead to fewer but higher-quality research papers being published and ultimately save time and resources down the road. Of course, an external mandate for greater rigor and accountability would also mean the need for funding agencies to financially support the extra effort and develop ways to monitor the labs' adherence to the new stricter rigor and dissemination practices, but it is commonsense that in the long run it is cheaper to do the experiment right the first time around than waste years trying to reproduce or follow up on erroneous data or remaking the resource that has been generated previously.

## Synthetic Biology

An exciting and highly promising area of sciences that plant biologists are starting to embrace more widely is synthetic biology. First, what is synthetic biology? To a plant biologist, it is a useful extension of classical molecular genetics that integrates basic engineering principles and aims to rebuild biology from the ground up. Traditionally, classically trained biologists approach learning about nature from top to bottom, much like a curious child trying to break a toy apart to see what it is made of. Synthetic biologists, vice versa, try to rebuild a functional system from its pieces to understand what its minimal required components are. In plant biology, we are still very far from being able to rebuild entire plants or plant cells from scratch, but we can reconstitute the pathways, e.g., those that we have previously studied in their native context, in a heterologous host cell, aka the chassis, or introduce simple gene regulatory circuits we have artificially built. Why would we want to do that? For one, to see if we can recreate the native behavior to ensure that we fully understand the pathway or the mechanism of regulation. In addition, this can be a useful endeavor from a practical perspective, as is the case in metabolic engineering, where a native or semi-synthetic biosynthetic pathway is expressed in a heterologous host (an intact plant or a cell suspension) to produce a valuable metabolite (Lu et al., 2016; Birchfield and McIntosh, 2020), or in biosensing, where a synthetic genetic construct is introduced to turn the host into a bio-detector for a particular stimulus or ligand of interest, e.g., a metabolite (Garagounis et al., 2021).

We do not fully comprehend what we cannot ourselves recreate. We may know, for example, that a gene is induced, for example, by heat stress, but that observation does not tell us anything about the developmental regulation of that gene, or what other biotic or abiotic factors control this gene's expression. An illustrative example of how limited our current knowledge is and how synthetic biology can help us to bypass the lack of comprehensive understanding is to try the following mental exercise. How would one go about conferring a desired pattern of expression to a gene of interest, so that the gene is transcribed, for example, only in a flower, in the anthers at a particular stage of flower development, and only in response to heat stress? If we are talking about a model organism, we can scavenge available transcriptomic data in hopes of finding a native gene with such a pattern, but chances are that most anther-enriched genes will be expressed elsewhere and/or will be regulated by stimuli other than the heat stress. With the vast amount of transcriptomic data and limited ChIP-seq, DAP-seq and chromatin availability data (ATAC-seq, DNase-seq, etc.), we still have no reliable ways to infer transcription patterns of a native gene across all tissues and conditions. A combination of bioinformatic analysis (to identify putative transcription factor binding sites based on sequence conservation) (Zemlyanskaya et al., 2021), classical transgene promoter bashing (that involves building a series of transgenes with chunks of the promoter deleted or replaced in an effort to characterize the effect of these targeted DNA modifications on the expression of a reporter gene in a systematic manner) (Andersson and Sandelin, 2020), and/or more recently, *in planta* promoter bashing via genome editing (i.e., generating targeted promoter modifications directly in the native genomic context) (Pandiarajan and Grover, 2018) are often relied upon to identify regulatory *cis*-elements in the promoters of interest. However, these approaches will not be enough to identify the full array of the DNA *cis*-elements that dictate the spatiotemporal regulation of a gene of interest, but these strategies may be helpful at pinpointing some candidate *cis*-elements and experimentally validating which elements are required.

**Figure 1:**
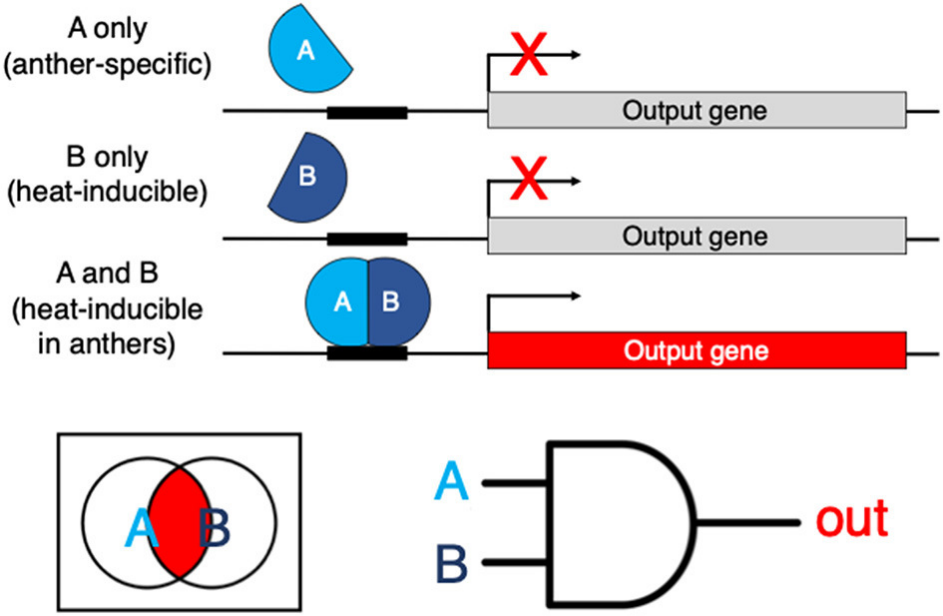
An example of a hypothetical genetic Boolean logic AND gate. AB is a heterodimeric transcription factor. If subunit A is expressed in anthers and subunit B is inducible by heat, the full transcription factor is reconstituted only in heat-stressed anthers. The AND logic restricts the expression of the output gene of interest specifically to the tissues and conditions where/when both A and B are-co-expressed.

If a particular DNA element is experimentally shown to be necessary, let's say, for heat stress upregulation, the next step is to test if the element is sufficient. This could be done by building a tandem of these elements, making a synthetic proximal promoter and placing it upstream of a well-characterized core promoter like that of *35S* to drive a reporter (Ali and Kim, 2019). In the best-case scenario, if we are successful with finding an element that can confer heat-inducible expression to the reporter, we have no easy way of restricting this heat-activated expression to just the anthers, let alone at a specific stage of anther development. Even if we had another DNA element at hand that confers tissue-specific expression (in this example, in anthers), we have no straightforward way of implementing what computer scientists would view as the Boolean AND logic—to combine these DNA elements (e.g., in a single proximal promoter) in a manner that the transcription of the gene will now only be triggered specifically in anthers in response to heat, but not in any other conditions or tissues. Synthetic biology makes the implementation of that AND logic (and other types of Boolean logic gates) possible, e.g., through the use of heterodimeric transcription factors, with one monomer active in anthers (through the use of an anther-specific promoter) and another monomer expressed only in response to heat stress (through the use of a heat-regulated promoter) (Figure 1). In this scenario, the full heterodimeric transcription factor would only be reconstituted in the anthers of heat-treated plants and will activate its target genes only in those flower tissues specifically under heat stress.

Thus, synthetic biology enables us to build genetic devices capable of controlling specific processes of interest despite the lack of the full mechanistic understanding of all the moving parts in those processes. In the near future, more and more plant biologists will adopt synthetic biology as a powerful way to bypass some of the technical bottlenecks in plant sciences. Who knows, someday futuristic concepts of a minimal plant genome and a minimal plant cell (Yang et al., 2020) may even become a reality. How soon will we have a thorough enough understanding of plant molecular genetics and physiology, so that we can determine the minimal set of genes to make a functional plant that can stay alive in a single stable (optimal) environment? What would we need to add to the minimal system to make the plant now capable of responding to stress and thriving in less-than-optimal conditions? Although one would agree that we have a very long way before we can get there, it is not too early to start thinking about those more ambitious projects, while working on still very difficult but more achievable shorter-term goals where synthetic biology will play a central role, such as developing nitrogen-fixing cereal crops (Bloch et al., 2020) or C4 rice (Ermakova et al., 2020).

## Other Directions and Concluding Remarks

Several other areas relevant to plant sciences will have paramount importance to our ability to propel plant biology research forward. Advanced automated high-throughput imaging and phenotyping will provide a more systematic, robust way to collect reliable morphometric data on a diversity of plant species in the lab, the greenhouse, and the field. New computational tool development and the implementation of novel experimental methods, along with the optimization and streamlining of existing tools and protocols, will remain the main driver of research progress, with single-cell omics approaches likely taking center stage for the next few years. Data science will play an even more predominant role given the vast amount of new data being generated and the need to handle and make sense of all that information. Systems-level approaches, mathematical modeling and machine learning will become a more integral part of plant biology research, enabling scientists to systematize and prioritize complex data and provide plant researchers with experimentally testable predictions.

If we want to see the breakthroughs we are making at the bench or in the field implemented in real-life products, we also need to work on shifting the public perception of biotechnologies. Critical steps toward rebuilding public trust in science include a greater understanding of the societal impacts of proposed innovations through collaboration with social scientists, the engagement of researchers with the science policy making process, and the active participation of all scientists (students, postdocs, technicians, faculty, industry professionals, etc.) in community outreach programs to make our work—and its implications—accessible to the general public. Lastly, one essential factor that would make the scientific advancements sustainable in the long run is a generous investment into the robust, trans-disciplinary training of the next generation of plant scientists. Our ability to create a welcoming environment for trainees from all backgrounds and paths of life would allow these students and postdocs to feel that their research team is their second family. Today's trainees are the ones who will be solving the world's pressing issues for years to come. Our ability to provide young scientists with the solid knowledge base and diverse skills would ensure that they are well equipped to take on the next big challenge.

Looking ahead, fundamental research on model organisms, applied work on crops, and conservation studies on rare plants will all continue to be of vital importance to modern plant biology. High-throughput inquiries and gene-specific projects done by mega-groups and small labs in state-of-the-art facilities or traditional field labs will all remain indispensable to the progress of plant sciences. In the end, addressing pressing societal issues like feeding the world's growing population and mitigating climate change ultimately rests on our ability as scientists to come together and harness the power of plants. Plant biology research is positioned to play a central role in this critical endeavor. It is an exciting and urgent time to be—or become—a plant scientist.

## Author Contributions

The author confirms being the sole contributor of this work and has approved it for publication.

## Funding

The work in the Stepanova lab is supported by the National Science Foundation grants NSF 1750006, NSF 1444561, NSF 1940829.

## Conflict of Interest

The author declares that the research was conducted in the absence of any commercial or financial relationships that could be construed as a potential conflict of interest.

## Publisher's Note

All claims expressed in this article are solely those of the authors and do not necessarily represent those of their affiliated organizations, or those of the publisher, the editors and the reviewers. Any product that may be evaluated in this article, or claim that may be made by its manufacturer, is not guaranteed or endorsed by the publisher.
